# Formation and Elimination of Satellite Droplets during Monodisperse Droplet Generation by Using Piezoelectric Method

**DOI:** 10.3390/mi12080921

**Published:** 2021-07-31

**Authors:** Zejian Hu, Shengji Li, Fan Yang, Xunjie Lin, Sunqiang Pan, Xuefeng Huang, Jiangrong Xu

**Affiliations:** 1 Institute of Energy, Department of Physics, Hangzhou Dianzi University, Hangzhou 310018, China; zejianhu@hdu.edu.cn (Z.H.); yf@hdu.edu.cn (F.Y.); 17072215@hdu.edu.cn (X.L.); jrxu@hdu.edu.cn (J.X.); 2 College of Materials and Environmental Engineering, Hangzhou Dianzi University, Hangzhou 310018, China; 3 Division of Biological and Chemical Metrology, Zhejiang Institute of Metrology, Hangzhou 310018, China; pansunqiang@hotmail.com

**Keywords:** monodisperse droplet generation, satellite droplets, piezoelectric method, droplet coalescence

## Abstract

One of the key questions in the generation of monodisperse droplets is how to eliminate satellite droplets. This paper investigates the formation and elimination of satellite droplets during the generation of monodisperse deionized water droplets based on a piezoelectric method. We estimated the effects of two crucial parameters—the pulse frequency for driving the piezoelectric transducer (PZT) tube and the volume flow rate of the pumping liquid—on the generation of monodisperse droplets of the expected size. It was found that by adjusting the pulse frequency to harmonize with the volume flow rate, the satellite droplets can be eliminated through their coalescence with the subsequent mother droplets. An increase in the tuning pulse frequency led to a decrease in the size of the monodisperse droplets generated. Among three optimum conditions (OCs) (OC1: 20 mL/h, 20 kHz; OC2: 30 mL/h, 30 kHz; and OC3: 40 mL/h, 40 kHz), the sizes of the generated monodisperse deionized water droplets followed a bimodal distribution in OC1 and OC2, whereas they followed a Gaussian distribution in OC3. The average diameters were 87.8 μm (OC1), 85.9 μm (OC2), and 84.8 μm (OC3), which were 8.46%, 6.14%, and 4.69% greater than the theoretical one (81.0 μm), respectively. This monodisperse droplet generation technology is a promising step in the production of monodisperse aerosols for engineering applications.

## 1. Introduction

In recent years, monodisperse droplet generation technology has been widely used in the fields of additive manufacturing [1,2], inkjet printing [3,4], electronic packaging [5,6], bioengineering [7,8,9], instrument calibration [10,11], etc. It has prompted many researchers to become engaged in developing droplet generation techniques to meet new requirements such as droplets that are highly uniform and monodisperse in terms of their size, shape, density, and surface characteristics, with a variety of solutes and solvents.

Many attempts have been made to generate monodisperse droplets, such as hot bubble [12], mechanical [13], pneumatic [14,15], piezoelectric [16], electromagnetic [17], and droplet-based microfluidic [18,19,20] technologies. Among these, the piezoelectric droplet generation method is one of the best choices to obtain monodisperse droplets. On the basis of the piezoelectric method, the generation of droplets depends on the control of the pulse waveform for the driving of the PZT tube. Li et al. [21] reported that monodisperse droplets can be obtained through adjusting the frequency and amplitude of a rectangular pulse waveform at a high operating pressure of 3.5 MPa. Fan et al. [22] reported that monodisperse droplets were generated by controlling the upper and lower limits of the pulse amplitude. However, during droplet generation, many satellite droplets were produced, leading to a wide size distribution of the droplets. Shin et al. [23] also reported that, for low viscosity liquid, satellites were generated when a single-pulse waveform was applied, whereas when a double-pulse waveform was utilized, the satellites were eliminated. A simple change in the time separation can precisely control the droplet size. If the time separation is shortened, the droplets’ size becomes smaller, since the second pulse reduces the mass and momentum of the ejected liquid. However, the double-pulse waveform can possibly result in the coalescence of two adjacent mother droplets. Lin et al. [24] further studied the influence of pulse frequency, positive voltage time, and voltage magnitude on droplet ejection velocity using a double-pulse voltage pattern, but they estimated it to have a scarce effect on droplet size.

Regardless of the control associated with the single-pulse waveform or the double-pulse waveform, the breakup of fluid filaments ejected from the nozzle leads to an array of uniformly spaced large droplets (called mother droplets) with smaller droplets (known as satellite droplets) in between them. Therefore, to generate uniform mother droplets based on piezoelectric method, the problem of eliminating a large number of satellite droplets has to be solved. Otherwise, this situation would result in a nonuniform size distribution of the breakup droplets. There is no doubt that one of the key steps involved in generating uniform and monodisperse droplets is eliminating the satellite droplets and avoiding the coalescence of two adjacent mother droplets.

In this work, we have aimed to investigate the formation and elimination of satellite droplets during the generation of monodisperse deionized water droplets based on the piezoelectric method. The emphasis of this study was on harmonizing the relationship between the frequency of the square-pulse waveform and the volume flow rate in order to obtain monodisperse droplets of the expected size. Therefore, this work involves: (1) observing the formation and elimination processes of satellite droplets during the generaton of monodisperse deionized water droplets through a high-resolution imaging system; (2) estimating the effect of pulse frequency and volume flow rate parameters on the droplet size and its distribution; (3) revealing the mechanisms involved in the generation and elimination of satellite droplets related to these two crucial parameters.

## 2. Experimental Methods

**Figure 1:**
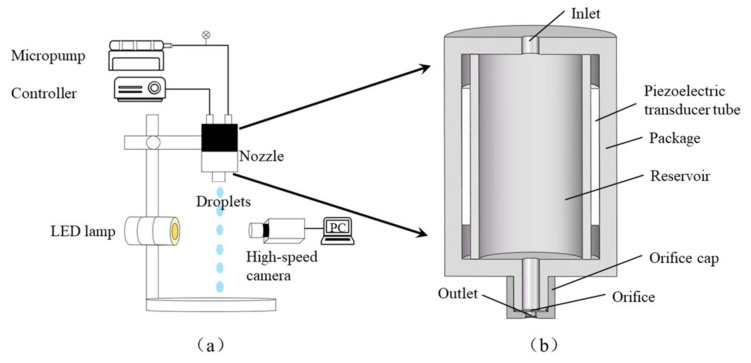
(**a**) Schematic of experimental setup; (**b**) schematic of nozzle.

A schematic of the experimental setup is shown in Figure 1a. It was mainly composed of a micropump and syringe, filter, nozzle, controller, high-speed camera, and an LED lamp. The micropump was used to feed the deionized water stored in the syringe and to control the volume flow rate with an accuracy of ± 2%. The deionized water (the properties of which are listed in Table 1) was transported to the nozzle through the connecting pipe and filter. The droplets were then extruded by means of the PZT tube in the nozzle, controlled by the controller. The high-speed camera (Phantom M310, Vision Research Inc., Wayne, NJ, USA) with a lens (AT-X M100 AF PRO, Tokina, Japan) was utilized to record the pinch-off process of the liquid filaments, the formation of mother droplets, and the generation of satellite droplets. The recording frame was set to 5000 fps. The LED lamp with an adjustable luminance was used to illuminate the field of view for clear imaging using the high-speed camera.

**Figure 2:**
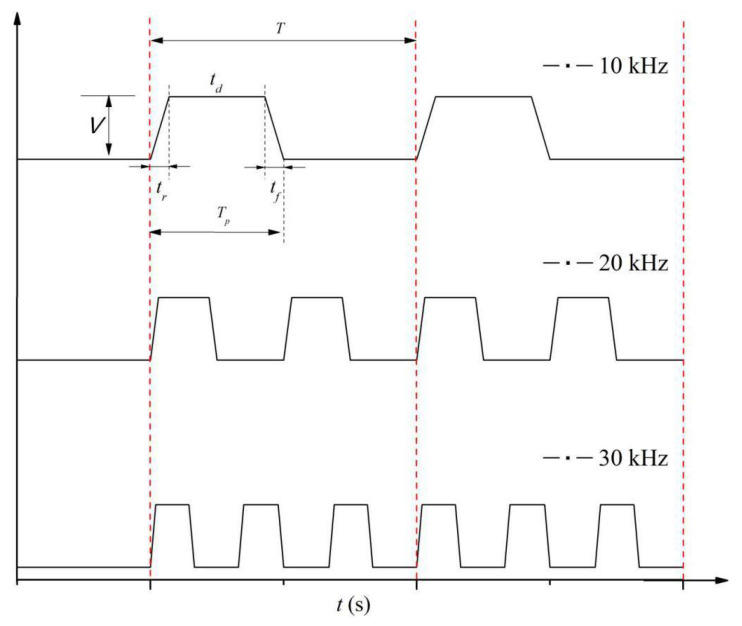
Schematic of periodic rectangle-wave pulse waveform.

The nozzle (MDG100) was purchased from TSI Co. Ltd., USA, and was based on a squeeze mode design. A PZT tube was wrapped outside a glass tubular reservoir, shown in Figure 1b. The orifice diameter at the bottom of the nozzle was 50 μm. The PZT tube was driven by the controller using a transistor–transistor logic (TTL) signal to modulate the width and frequency of periodic rectangle-wave pulse as shown in Figure 2. The period of the pulse waveform is *T*, and its voltage amplitude is *V*. The duty ratio is *T_p_*/*T*, where *T_p_* is the applied time of the pulse. The single-pulse waveform is a rectangle wave, which is defined by a pulse width, a rising edge, and a falling edge. The applying time of the pulse can be divided into three parts—the rising time (*t_r_*), dwelling time (*t_d_*), and falling time (*t_f_*). When the rising (falling) edge of the waveform is applied to the PZT tube, a negative (positive) pressure wave is produced, expanding (contracting) the glass tubular reservoir, so that the fluid filaments will be ejected and broken up. These parameters of the driving pulse for the PZT tube are listed in Table 2.

The measurement of droplet size is crucial to estimate performance in the generation of monodisperse droplets. In this work, a digital image processing method was used. The elaborate operation procedures have been presented in our previous work [25]. Briefly, (1) a magnified image of the calibrating ruler was acquired; (2) pictures of the breakup droplets were taken under the same imaging conditions; (3) a self-programming digital imaging treatment program was then used to compare the images of the breakup droplets with calibrated pixel pitches. If the droplets appeared ellipsoidal or non-spherical due to deformation, a characteristic dimension *d*_21_ (called the equivalent diameter) was calculated by *d*_21_ = 4*S_d_*/*L_d_*, where *S_d_* and *L_d_* represent the area and the perimeter of each droplet, respectively. Finally, the statistical average diameter of multiple droplets (*d*) was determined by the equation $d=\frac{\sum n_{i}d_{i}}{\sum n_{i}}$.

## 3. Results and Discussion

### 3.1. Pinch-Off of Liquid Filament and Generation of Satellite Droplets

**Figure 3:**
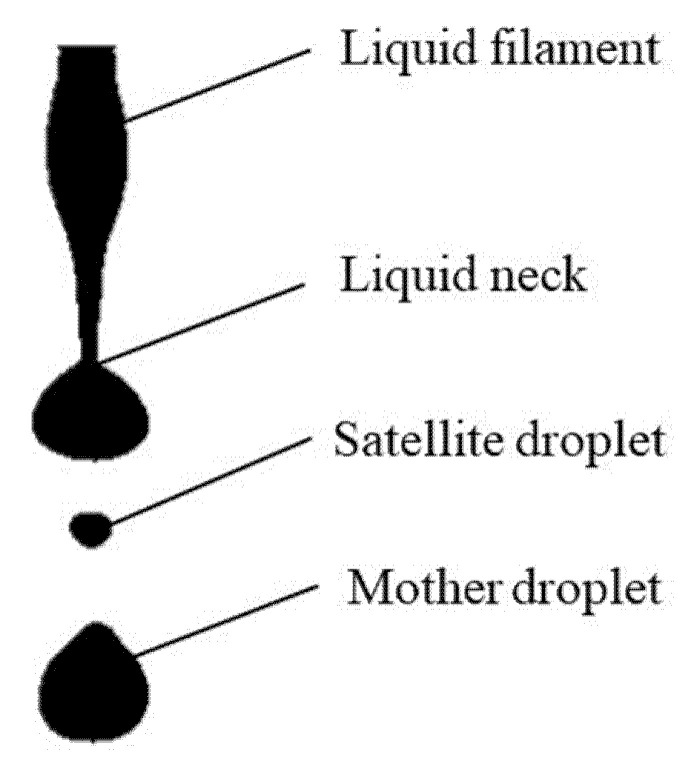
Snapshot of the pinch-off of a deionized water filament and the generation of satellite droplets.

Figure 3 demonstrates a snapshot taken during the pinch-off process of the liquid filament at a pulse frequency of 10 kHz and a volume flow rate of 30 mL/h. It can be observed that surface waves cover the liquid filament, and a mother droplet has been produced; another droplet is being generating at the liquid neck (the narrowest position along the liquid filament). At the bottom of the picture, when a mother droplet is detached from the liquid filament, a satellite droplet is simultaneously generated.

The Reynolds and Weber numbers can be expressed as *Re* = *ρvd/μ* and *We* = *ρv^2^d/σ*, in which *ρ*, *v*, *d*, *σ* and *μ* represent the density, velocity, orifice diameter, surface tension, and viscosity of the deionized water, respectively. In this case, the Reynolds number was ~186.2 and the Weber number was ~1.4. This suggests that the pinch-off of the deionized water filament generally exhibited a laminar regime, and the surface tension played a key role in the detachment.

The evolution of the liquid filament was considered as a function of the viscosity ratio (*p*) of the fluids and the initial wavenumber of the interface perturbation, and the satellite droplets were generated around the neck region of a highly deformed filament. Tjahjadt et al. [26] numerically and experimentally explained that, in low-viscosity ratio systems, *p* < *O* (0.1), the breakup mechanism depends on self-repetition in multiple breakup sequences in which every pinch-off is always associated with the formation of a neck; the neck undergoes pinch-off, and the process repeats. In this case, unlike multiple breakup sequences, at each period, we observed one breakup sequence and a generated satellite droplet. This means that, at each corresponding period of the controlled rectangle-wave pulse of PZT tube, the duty ratio, the frequency, and the sequence of time applied will be crucial in order to affect the formation of mother and satellite droplets.

### 3.2. Elimination of Satellite Droplets

Theoretically, if the liquid filament breaks up into monodisperse mother droplets without the generation of satellite droplets, the input volume per unit time should be equal to the total volume of the monodisperse droplets (mother droplets) generated per unit time:

(1) $$q\times \frac{10^{-6}}{3600}=\frac{\pi }{6}d_{th}^{3}\times 10^{-18}\times f\times 10^{3}$$

In which *d_th_*, *q*, and *f* represent the theoretical average diameter of the generated monodisperse droplets (μm), the volume flow rate of the feeding liquid (mL/h), and the pulse frequency for the driving of the PZT tube (kHz), respectively.

Thus, the theoretical average droplet diameter of the generated monodisperse droplets in an ideal state can be calculated as follows:

(2) $$d_{th}=\sqrt[{3}]{\frac{q}{600\pi f}}\times 10^{3}$$

**Figure 4:**
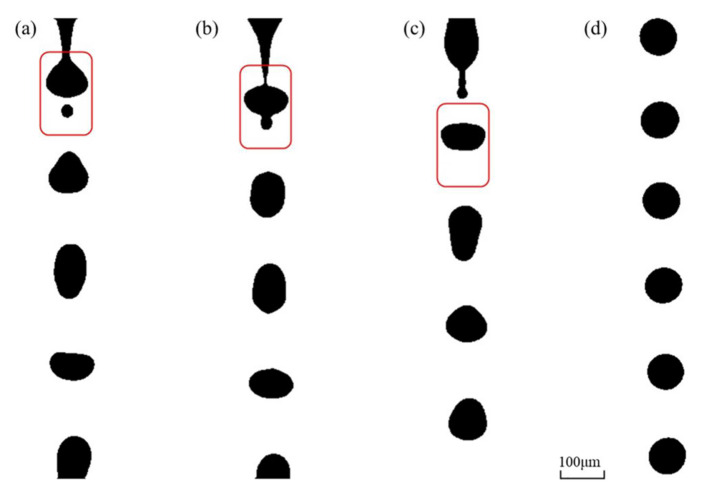
Snapshots of the pinch-offs of liquid filaments and the elimination of satellite droplets. (**a**) Formation of satellite droplets. (**b**,**c**) Satellite droplets merged by a subsequent mother droplet and eliminated; (**d**) a highly uniform size distribution of monodisperse droplets were obtained (volume flow rate of 30 mL/h; pulse frequencies of 30 kHz).

The volume flow rate of deionized water was still set to 30 mL/h. As the pulse frequency was adjusted from 10 kHz to 30 kHz, we observed the coalescence of satellite droplets and mother droplets, as shown in Figure 4. During the pinch-off process of the liquid filament, a satellite droplet was generated (Figure 4a), but it immediately merged with the subsequent mother droplet (Figure 4b,c). Finally, monodisperse droplets were generated with a highly uniform size distribution (Figure 4d). The average diameter of the generated monodisperse droplets was 85.9 μm in this case, which is 6.14% larger than the theoretical one. This suggests that the adjustment of the pulse frequency could eliminate the satellite droplets by means of coalescence, validating the feasibility of this methodology.

**Figure 5:**
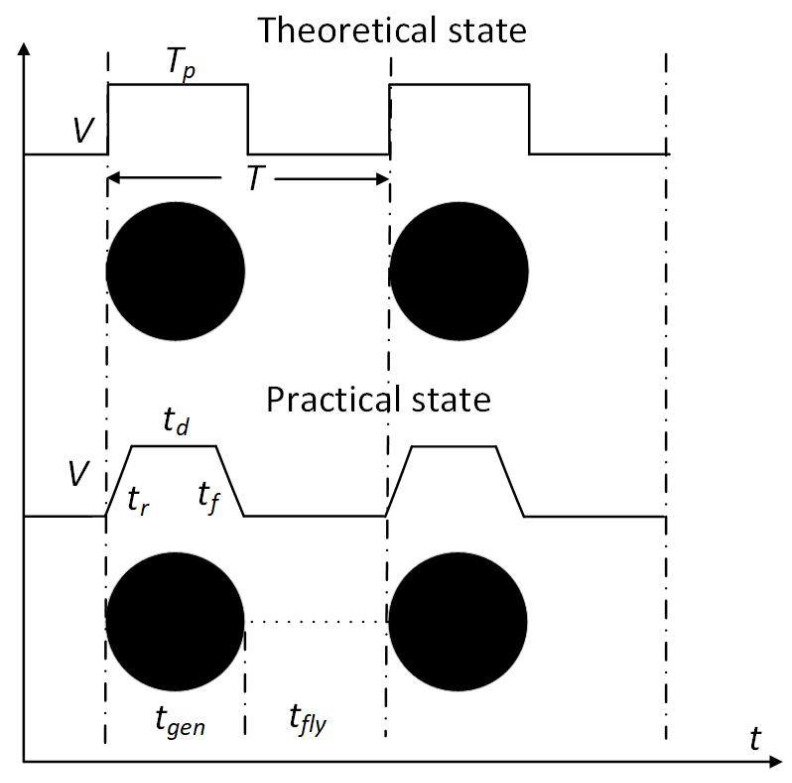
Schematic of the theoretical and practical generation of monodisperse droplets.

It is worthy of notice that the number of monodisperse droplets is theoretically equal to the pulse frequency, i.e., only a single droplet is generated as a pulse is applied to the PZT tube. Thus, the pulse width and the duty ratio of each pulse need to be negotiated for the detachment of each droplet from the liquid filament, as shown in Figure 5. The generation time of monodisperse droplets (*t_gen_*) is equal to the theoretical pulse width, or the applied time of the practical pulse, i.e., *t_gen_*=*T_p_*=*t_r_* + *t_d_* + *t_f_*, in which *t_r_*, *t_d_*, and *t_f_* are the rising time, dwelling time, and falling time, respectively. The flight time of the monodisperse droplets (*t_fly_*) is the same as the vacant time of the pulse, i.e., *t_fly_*=*T* − *T_p_*, in which *T* and *T_p_* are the period of the pulse waveform and the applied time of the pulse, respectively. Lin et al. [24] suggested that a sufficient time of *t_r_* and *t_f_* is necessary to reach the desired voltage amplitude and to make the PZT tube expand and contract enough. The empirical expression of the voltage amplitude of the pulse waveform must meet the following requirements [24]:

(3) $$V/t_{r}\leq 15,V/t_{f}\leq 15$$

This indicates that the slope of the voltage variation should be less than 15 V/μs. Additionally, the voltage amplitude should be high enough to ensure that the droplet is ejected, but not so high that the ejection becomes chaotic and it becomes difficult to obtain monodisperse droplets.

To generate monodisperse droplets, the rising time (*t_r_*) and the falling time (*t_f_*) should also harmonize with the detaching time of each droplet. Under the same operating conditions, the detaching time depends mainly on the viscosity of the liquid. The larger the viscosity, the longer the detaching time becomes. The dwelling time (*t_d_*) has been given by Bogy et al. [27], i.e., the optimum pulse width (*t_opt_*), which can be calculated as follows:

(4) $$t_{d}=t_{opt}=L/v_{aco,liq}$$

in which *L* and *v_aco,liq_* are the length of nozzle and the velocity of acoustic wave propagation in the liquid, respectively. In this work, the theoretical value of the optimum dwelling time (*t_d_*) was ~12 μs. In Figure 4d, the central spacing between two adjacent droplets was twice as large as the droplet diameter, since the duty ratio of the rectangular pulse was 0.5. To avoid the coalescence of two spherical mother droplets without satellite droplets, the central spacing must be greater than the droplet diameter. However, since the droplets exhibited severe deformation after being detached from the liquid filament (Figure 4a–c), the maximum ratio of droplet length to width reached 1.8, and the central spacing was close to the droplet diameter. This suggests that the duty ratio should be below 0.8.

### 3.3. Effect of Pulse Frequency on the Elimination of Satellite Droplets

**Figure 6:**
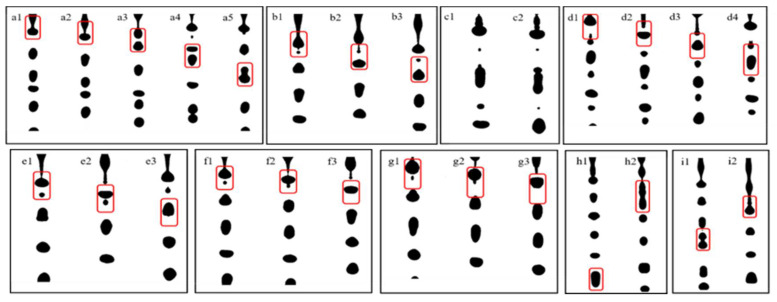
Snapshots of droplet generation at the volume flow rate of 30 mL/h at different pulse frequencies ((**a1**–**a5**): 5 kHz; (**b1**–**b3**): 10 kHz; (**c1**–**c2**): 15 kHz; (**d1**–**d4**): 20 kHz; (**e1**–**e3**): 25 kHz; (**f1**–**f3**): 30 kHz; (**g1**–**g3**): 35 kHz; (**h1**–**h2**): 40 kHz; (**i1**–**i2**): 45 kHz).

Figure 6 demonstrates the snapshots of droplet generation as the pulse frequency ranged from 5 to 45 kHz at the volume flow rate of 30 mL/h. At the pulse frequencies of 5 kHz, 10 kHz, and 15 kHz, it was observed that the satellite droplets were difficult to eliminate (Figure 6b3,c2), and the two adjacent mother droplets coalesced (Figure 6a5). When the pulse frequency was enhanced to 20 kHz or 25 kHz, satellite droplets with greater size were generated (Figure 6d4,e3). At 30 kHz and 35 kHz, the satellite droplets were almost eliminated (Figure 6f3,g3). However, as the pulse frequency exceeded 40 kHz, the generated droplets were chaotic, and they had a wide size distribution.

Experiments on the influence of pulse frequency on the formation and elimination of satellite droplets were also carried out at the volume flow rates of 20 mL/h and 40 mL/h, respectively. According to the results, it can be concluded that, at a certain volume flow rate, the pulse frequency must be adjusted to an optimum range to generate monodisperse droplets. At the volume flow rate of 20 mL/h, the optimum range of the pulse frequency was 18–22 kHz. For the volume flow rates of 30 mL/h and 40 mL/h, the optimum ranges of the pulse frequency were 25–35 kHz and 35–50 kHz, respectively.

### 3.4. Effect of Pulse Frequency on the Average Diameter of Droplets

**Figure 7:**
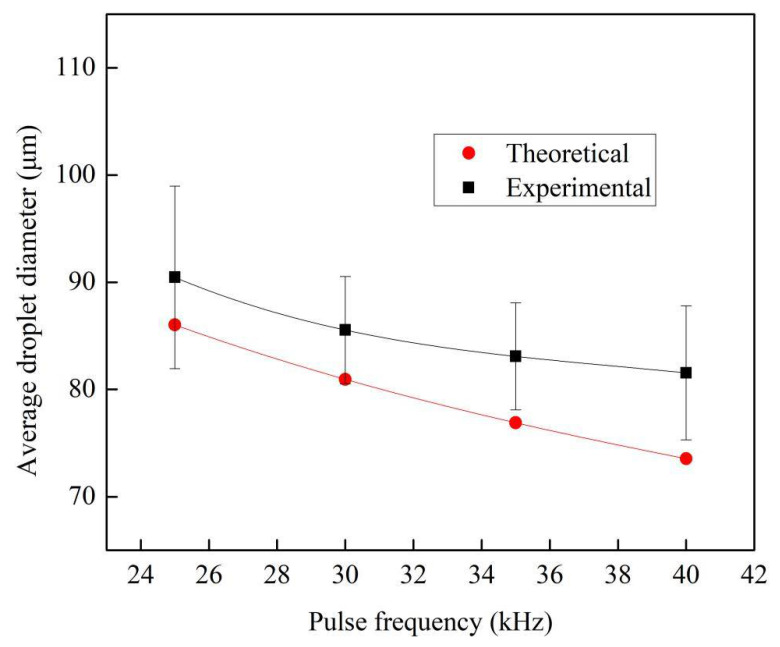
Average droplet diameters at the volume flow rate of 30 mL/h at different pulse frequencies of 25 kHz–40 kHz.

According to Equation (2), the diameter of the generated droplets depends on the volume flow rate and the pulse frequency. As the volume flow is fixed, the desired diameter of the droplets can be obtained through adjusting the pulse frequency. Figure 7 shows the average diameters of the generated droplets at the volume flow rate of 30 mL/h, at different pulse frequencies of 25–40 kHz, with an interval of 5 kHz. The theoretical diameter of the droplets was calculated using Equation (2), and these are also shown in Figure 7. In general, the experimental result displayed a similar trend to the theoretical one. As the pulse frequency for driving the PZT tube was enhanced, the average diameter of the generated droplets decreased. The average droplet diameters based on the experiments were 5–11% greater than those based on the theoretical calculation. This difference can be attributed to the random error associated with dealing with the images and the systematic calibration error.

### 3.5. Size Distribution of Monodisperse Droplets under Optimum Operating Conditions

**Figure 8:**
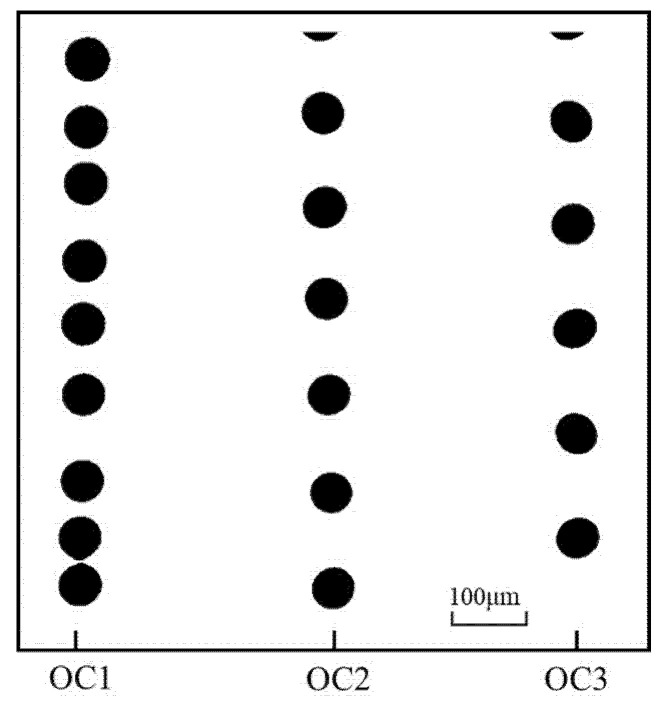
Snapshots of monodisperse droplets generated under three optimum conditions (OC1: 20 mL/h, 20 kHz; OC2: 30 mL/h, 30 kHz; and OC3: 40 mL/h, 40 kHz).

If the ratio of the volume flow rate and the optimum pulse frequency are kept constant, based on Equation (2), the diameter of the generated droplet would be theoretically the same. This was also supported by the experimental results. Figure 8 illustrates the snapshots of the generated monodisperse droplets under three optimum conditions (OCs) (OC1: 20 mL/h, 20 kHz; OC2: 30 mL/h, 30 kHz; and OC3: 40 mL/h, 40 kHz). In the optimum pulse frequency range, no satellite droplets were observed.

**Figure 9:**
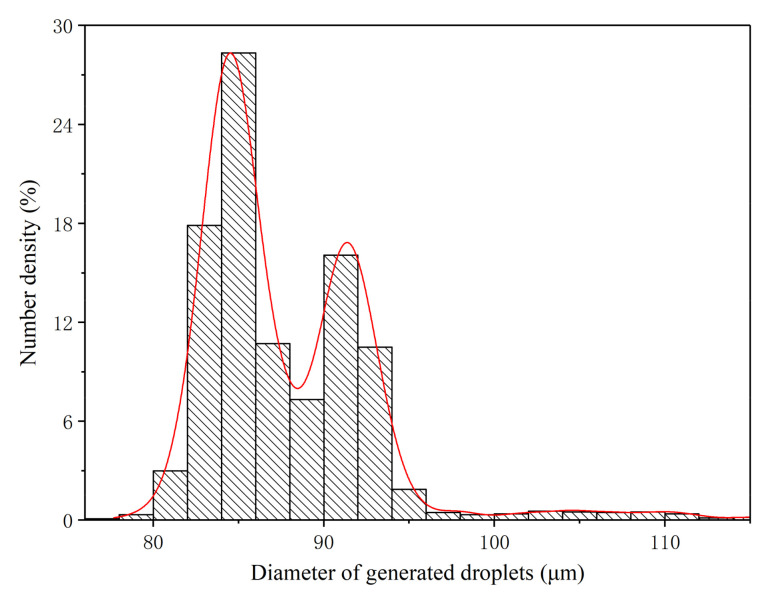
Size distribution of generated monodisperse droplets under OC1 (*q* = 20 mL/h, *f* = 20 kHz).

**Figure 10:**
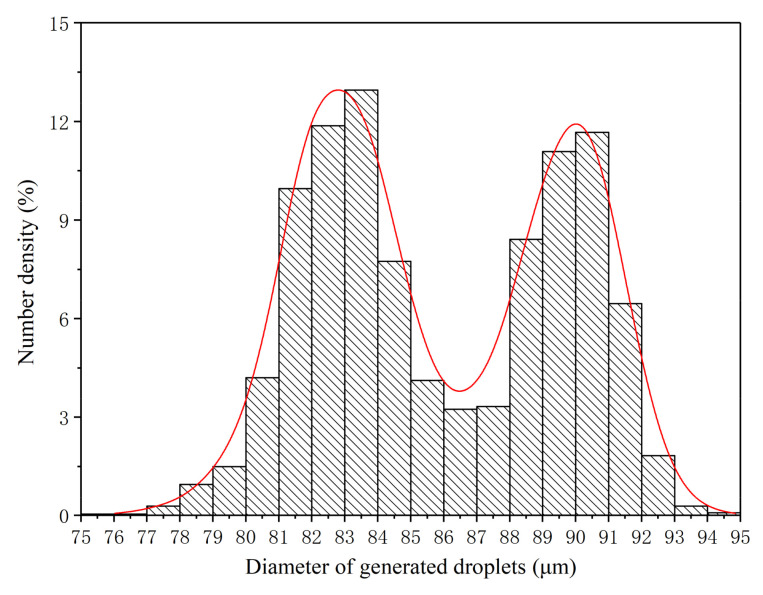
Size distribution of generated monodisperse droplets under OC2 (*q* = 30 mL/h, *f* = 30 kHz).

**Figure 11:**
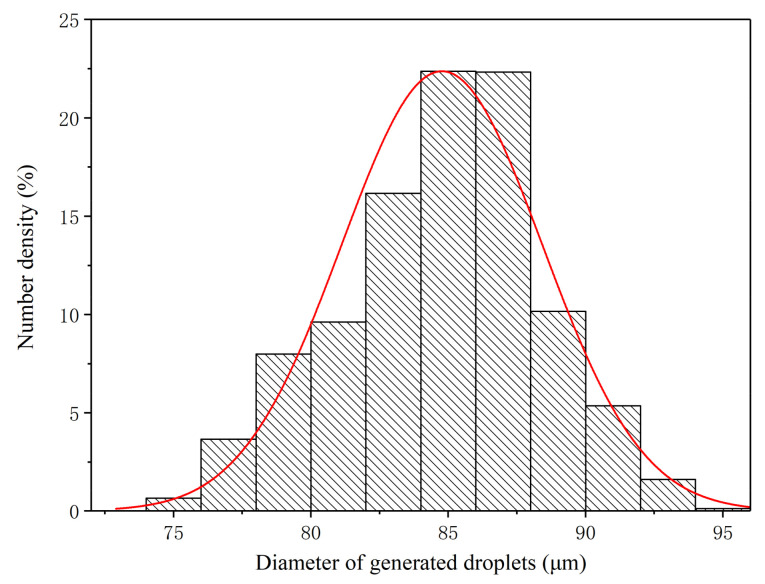
Size distribution of generated monodisperse droplets under OC3 (*q* = 40 mL/h, *f* = 40 kHz).

The size distributions of the monodisperse droplets generated under the three optimum conditions were further extracted and analyzed in detail, as shown in Figure 9, Figure 10 and Figure 11. Under OC1, the size of generated monodisperse droplets were mainly distributed in the range of 80–95 μm, with a small number of droplets of 95–110 μm (Figure 9). As for OC2, the size distribution of generated monodisperse droplets ranged from 80 to 93 μm (Figure 10). In OC3, the droplet size was mainly distributed in the range of 75–95 μm (Figure 11). We observed two-peak profiles for both OC1 and OC2. Compared with the bimodal size distribution in OC1 and OC2, under OC3, the generated monodisperse droplets had a better dispersion uniformity, and the droplet size followed a Gaussian distribution.

**Figure 12:**
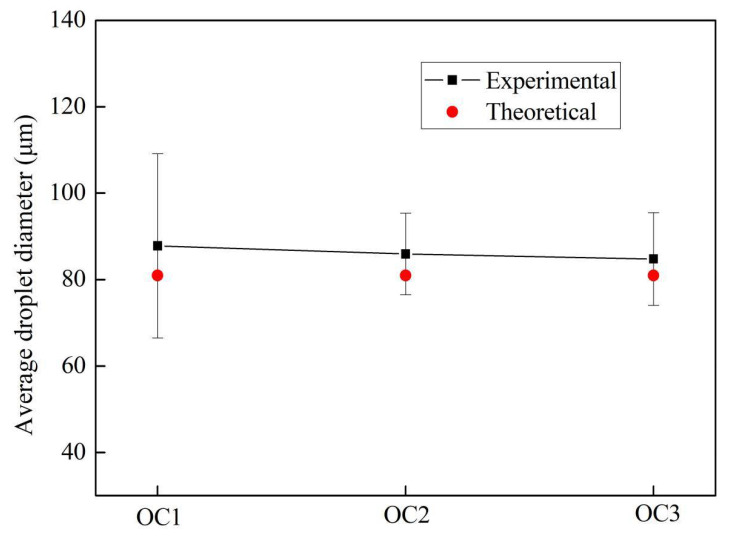
Average diameters of theoretically and experimentally generated monodisperse droplets.

The droplets generated under the three optimum conditions were generally well dispersed and had a good uniformity of dispersion. The average diameters of the generated monodisperse droplets were 87.8 μm (OC1), 85.9 μm (OC2), and 84.8 μm (OC3), respectively, as shown in Figure 12. Compared to the theoretical diameter (81.0 μm), the experimental results were 8.46%, 6.14%, and 4.69% greater than the theoretical one, respectively.

## 4. Conclusions

Experiments on the generation of monodisperse deionized water droplets based on the piezoelectric method were conducted. Our observations demonstrated that satellite droplets were generated because the mother droplets detached from the liquid filament with surface waveform perturbations. As the frequency of the square-wave pulse was tuned to match the volume flow rate of the pumping liquid, the satellite droplets merged with the subsequent mother droplets, and we were thus able to generate monodisperse droplets with a uniform size distribution. If the pulse frequency is relatively low, the satellite droplets cannot merge with the subsequent mother droplets. It is also possible that the two adjacent mother droplets can coalesce if the pulse frequency is so high that the generated droplets are chaotic. This results in a wide size distribution. Under different volume flow rates, the optimal ranges of pulse frequency required to generate monodisperse droplets are different. The size of the monodisperse droplets decreases with the increase in the pulse frequency, as the volume flow rate is given. The sizes of the monodisperse droplets generated follow bimodal and Gaussian distributions under optimum operating conditions.

## Figures and Tables

**Table 1:** Physical properties of deionized water.

Items	Density (kg/m^3^)	Surface Tension (N/m) @25 °C	Viscosity (Pa·s) @15 °C	Acoustic Velocity (m/s)
**Value**	1.0 × 10^3^	0.072	1.14 × 10^−3^	1435 [24]

**Table 2:** Parameters of pulse waveform for driving the PZT tube.

Items	Amplitude	Frequency	Duty Ratio
**Value**	4.2 V	5~40 kHz	0.5

## References

[1] Yamaguchi K., Sakai K., Yamanaka T. (2000). Generation of Three-Dimensional Micro Structure Using Metal Jet. Precis. Eng..

[2] Behera D., Cullinan M. (2021). Current Challenges and Potential Directions Towards Precision Microscale Additive Manufacturing—Part I: Direct Ink Writing/Jetting Processes. Precis. Eng..

[3] Zeng H., Yang J., Katagiri D., Ying R., Xue S., Nakajim H., Uchiyama K. (2015). Investigation of Monodisperse Droplet Generation in Liquids by Inkjet. Sensor. Actuat. B Chem..

[4] Kim C.S., Park S.J., Sim W. (2009). Modeling and Characterization of an Industrial Inkjet Head for Micro-Patterning on Printed Circuit Boards. Comput. Fluids.

[5] Carter J.C., Alvis R.M., Brown S.B. (2006). Fabricating optical Fiber Imaging Sensors Using Inkjet Printing Technology: A pH Sensor Proof-Of-Concept. Biosens. Bioelectron..

[6] Liu Q., Orme M. (2001). High Precision Solder Droplet Printing Technology and the State-Of-The-Art. J. Mater. Process. Technol..

[7] Hossain S., Luckham R.E., Smith A.M. (2009). Development of a Bioactive Paper Sensor for Detection of Neurotoxins Using Piezoelectric Inkjet Printing of Sol? Gel-Derived Bioinks. Anal. Chem..

[8] Sackmann E.K., Fulton A.L., Beebe D.J. (2014). The Present and Future Role of Microfluidics in Biomedical Research. Nat. Cell Biol..

[9] Hochstetter A. (2020). Lab-on-a-Chip Technologies for the Single Cell Level: Separation, Analysis and Diagnostics. Micromachines.

[10] Delene D.J., Deshler T. (2000). Calibration of a Photometric Cloud Condensation Nucleus Counter Designed for Deployment on a Balloon Package. J. Atmos. Ocean. Technol..

[11] Kalantarifard A., Alizadeh-Haghighi E., Saateh A., Elbuken C. (2021). Theoretical and Experimental Limits of Monodisperse Droplet Generation. Chem. Eng. Sci..

[12] Chen J., Wise K.D. (1997). A High-Resolution Silicon Monolithic Nozzle Array for Inkjet Printing. IEEE Trans. Electron Devices.

[13] Jadamson S., Manager S.P., Wang A.D. (2004). A Change in Dispensing Technology-Jetting Takes Off. Semicond. Tech..

[14] Cheng S., Chandra S. (2003). A Pneumatic Droplet-On-Demand Generator. Exp. Fluids.

[15] Cheng S.X., Li T., Chandra S. (2005). Producing Molten Metal Droplets with a Pneumatic Droplet-On-Demand Generator. J. Mater. Process. Technol.

[16] Sun J.M., Wei X.F., Huang B.Q. (2012). Influence of the Viscosity of Edible Ink to Piezoelectric Ink-Jet Printing Drop State. Appl. Mech. Mater..

[17] Tseng A.A., Lee M.H., Zhao B. (2001). Design and Operation of a Droplet Deposition System for Freeform Fabrication of Metal Parts. J. Eng. Mater. Tech..

[18] Teo A.J.T., Li K.-H.H., Nguyen N.-T., Guo W., Heere N., Xi H.-D., Tsao C.-W., Li W., Tan S.H. (2017). Negative Pressure Induced Droplet Generation in a Microfluidic Flow-Focusing Device. Anal. Chem..

[19] Filatov N.A., Evstrapov A.A., Bukatin A.S. (2021). Negative Pressure Provides Simple and Stable Droplet Generation in a Flow-Focusing Microfluidic Device. Micromachines.

[20] Chung C.H.Y., Cui B., Song R., Liu X., Xu X., Yao S. (2019). Scalable Production of Monodisperse Functional Microspheres by Multilayer Parallelization of High Aspect Ratio Microfluidic Channels. Micromachines.

[21] Li H., Liu F., Li Y. (2007). The Characteristics of 20 μm-Diameter Single Droplets. Acta Phys. Sin..

[22] Fan K.C., Chen J.Y., Wang C.H. (2008). Development of Drop-On-Demand Droplet Generator for One-Drop-Filling Technology. Sens. Actuator A Phys..

[23] Shin P., Sung J. The Effect of Driving Waveforms on Droplet Formation in a Piezoelectric Inkjet Nozzle. Proceedings of the 2009 11th Electronics Packaging Technology Conference.

[24] Lin H., Wu H., Shan T. (2006). The Effects of Operating Parameters on Micro-Droplet Formation in a Piezoelectric Inkjet Printhead Using a Double Pulse Voltage Pattern. Mater. Trans..

[25] Li S., Zhuo Z., He L., Huang X. (2019). Atomization Characteristics of Nano-Al/Ethanol Nanofluid Fuel in Electrostatic Field. Fuel.

[26] Tjahjadi M., Stone H.A., Ottino J.M. (1992). Satellite and Subsatellite Formation in Capillary Breakup. J. Fluid Mech..

[27] Bogy D.B., Talke F.E. (1984). Experimental and Theoretical Study of Wave Propagation Phenomena in Drop-On-Demand Ink Jet Devices. IBM J. Res. Dev..

